# Early Introduction of Food Allergens and Risk of Developing Food Allergy

**DOI:** 10.3390/nu13072318

**Published:** 2021-07-05

**Authors:** Elizabeth Yakaboski, Lacey B. Robinson, Anna Arroyo, Janice A. Espinola, Ruth J. Geller, Ashley F. Sullivan, Susan A. Rudders, Carlos A. Camargo

**Affiliations:** 1 Division of Rheumatology, Allergy and Immunology, Massachusetts General Hospital, Boston, MA 02114, USA; eyakaboski@mgh.harvard.edu (E.Y.); lbrobinson@mgh.harvard.edu (L.B.R.); 2 Division of Pulmonary, Allergy and Critical Care Medicine, Stanford University School of Medicine, Stanford, CA 94305, USA; 3 Department of Emergency Medicine, Massachusetts General Hospital, Boston, MA 02114, USA; ESPINOLA@helix.mgh.harvard.edu (J.A.E.); rjgeller@mgh.harvard.edu (R.J.G.); afsullivan@partners.org (A.F.S.); 4 Division of Immunology, Boston Children’s Hospital, Boston, MA 02115, USA; susan.rudders@childrens.harvard.edu

**Keywords:** early food allergen introduction, egg allergy, peanut allergy, food allergy, food hypersensitivity, oral tolerance

## Abstract

There is increasing evidence that early introduction of allergenic foods may decrease the risk of developing IgE-mediated food allergy. Patterns of food introduction before the 2015 publication of the Learning Early about Peanut Allergy (LEAP) trial are not well-studied, but are important as a baseline for evaluating subsequent changes in infant feeding practices and potentially food allergy. We performed a retrospective longitudinal study using data from a multicenter cohort of infants hospitalized with bronchiolitis between 2011–2014. The primary outcomes were IgE-mediated egg or peanut allergy by age 3 years. Of 770 participants included in the analysis, 635 (82%) introduced egg, and 221 (27%) introduced peanut by age 12 months per parent report. Four participants had likely egg allergy, and eight participants had likely peanut allergy by age 3 years. Regular infant egg consumption was associated with less egg allergy. The association was suggestive for infant peanut consumption with zero peanut allergy cases. Overall, our results suggest that early introduction of peanut was uncommon before 2015. Although limited by the small number of allergy cases, our results suggest that early introduction of egg and peanut are associated with a decreased risk of developing food allergy, and support recent changes in practice guidelines.

## 1. Introduction

The prevalence of IgE-mediated food allergy is rising, and is currently estimated to affect approximately 5–10% of children in the United States (US) [1,2,3]. To address this growing public health burden, strategies for prevention of food allergy development are of great interest [4,5,6,7]. In addition, to prevent accidental food allergen exposure in both children and adults with known food allergies, several recent industry advances in allergen detection and removal from processed foods have emerged [8].

Approaches for prevention of food allergy development that have not yielded reproducible results include prenatal maternal dietary avoidance, breastfeeding, the use of extensively hydrolyzed formulas, and pro- and prebiotics [4]. While there is emerging evidence that optimizing eczema care to maintain a healthy skin barrier and thus prevent food-specific sensitization likely plays an important role in food allergy prevention [9,10], the approach backed by the most convincing data is in regard to early introduction of allergenic foods, most notably egg and peanut [11,12,13,14,15,16,17,18].

Of these studies, the landmark clinical trial Learning Early about Peanut Allergy (LEAP), published in 2015, marked the beginning of a shift in clinical practice towards early introduction of food allergens. In this study, a cohort of infants aged 4 to 10 months with egg allergy, severe eczema, or both, were randomly assigned to consume or avoid age-appropriate peanut-containing foods several times per week up until age 5 years. The study did not include infants who already had peanut allergy. The LEAP study showed that early introduction of peanut decreased the prevalence of IgE-mediated peanut allergy at age 5 years by more than 80% [11]. In a follow-up two-group comparison study, the Persistence of Oral Tolerance to Peanut (LEAP-On) study showed that participants who introduced peanuts in infancy and continued to consume peanuts until 5 years of age, but then subsequently avoided peanut from age 5 to 6, were still 74% less likely to have peanut allergy than children who had consistently avoided peanut from infancy up until age 6. LEAP-On showed that the peanut tolerance promoted by early introduction to peanut could be long lasting [12].

Recent guidelines, including the 2021 multi-society Consensus document on approach to the primary prevention of food allergy [19], recommend early introduction of egg-containing and peanut-containing products starting around 6 months of age (but not before 4 months of age) in all infants, and to not deliberately delay the introduction of other potentially allergenic complementary foods. Similarly, the 2008 American Academy of Pediatrics Clinical Report was updated in 2019 and the 2010 National Institute of Allergy and Infectious Diseases Guidelines were addended in 2017 to recommend the same for peanut, with the caveat that infants at high risk for food allergy undergo evaluation for preexisting peanut sensitization prior to introduction [20,21,22,23,24]. Clinically, evaluation for pre-existing sensitization may be performed through skin-prick-testing and/or through ImmunoCAP specific-IgE either to whole food allergens or to food allergen components. While there is not a standardized definition of high risk for food allergy, the 2021 Consensus document recommends that infants with severe eczema are considered at highest risk of developing food allergy, and infants with mild to moderate eczema, parent(s) with a history of atopy, and a known food allergy have an elevated risk of developing a new food allergy. Although the 2014 food allergy prevention guidelines from the European Academy of Allergy and Clinic Immunology (EAACI)] stated that at the time of its publication, there was insufficient evidence to recommend either withholding or encouraging exposure to potentially allergenic foods after 4 months of age in all infants, with and without atopic heredity [25], the 2020 updated guidelines now suggest introducing peanut and well-cooked egg as part of complementary feeding [26].

Patterns of food introduction before the 2015 LEAP publication (and adoption of early allergen introduction), are not well-studied, but are important as a baseline for future changes in food introduction patterns. Our objectives were to first describe the patterns of egg and peanut introduction prior to 12 months of life in a US-based cohort and to then estimate the associations between early introduction of egg or peanut and early childhood IgE-mediated allergy to egg or peanut, respectively. To determine egg and peanut allergy status, allergy physicians reviewed data from multiple sources including primary medical records, serial interviews starting at age 12 months, as well as egg and peanut IgE obtained during infancy and at 3 years of age.

## 2. Materials and Methods

### 2.1. Study Population

We analyzed data from the ongoing 35th Multicenter Airway Research Collaboration (MARC-35), a multicenter, prospective cohort of infants enrolled during hospitalization for bronchiolitis during the fall/winter seasons of 2011–2014. MARC-35 is coordinated by the Emergency Medicine Network (EMNet; www.emnet-usa.org). MARC-35 was designed with the primary purpose of studying severe bronchiolitis and recurrent wheezing in early childhood, and also entailed the collection of a substantial amount of food allergy data for secondary studies such as the one presented here. The total number of sites participating was 17, across 14 US states. Full details of initial study inclusion and methods have been previously described [27,28].

### 2.2. Data Collection

Upon enrollment during infancy, investigators completed a structured interview with parents/guardians to assess patients’ demographic characteristics and medical and environmental history. Telephone interviews were then conducted every 6 months by the EMNet Coordinating Center at Massachusetts General Hospital (Boston, MA, USA), and included annual questions regarding dietary intake. Further clinical data on the patient’s clinical course, including medical records from all primary care and specialty medical visits, were obtained. Records were reviewed by physician reviewers and study data were collected and managed using Research Electronic Data Capture (REDCap) hosted at Mass General Brigham. REDCap is a secure, web-based application designed to support data capture for research studies [29,30]. IgE sensitization to common food allergens (e.g., egg and peanut) were assessed via ImmunoCAP Specific-IgE (sIgE) and immuno-solid-phase allergen chip (ISAC) both at the Phadia Immunology Reference Laboratory (Portage, MI, USA) during infancy and the early childhood examination (Table S1). Both are used to detect allergenic sensitization, but sIgE is considered the gold standard for in vitro diagnosis.

### 2.3. Exposure—Age 12 Months

Early introduction of egg or peanut was defined as consumption of egg product or peanut product, respectively, at least once per week based on parent report at the 12-month telephone follow-up interview. Specifically, parents were asked, did the participant eat or drink any of the following items at least once per week: (1) egg or egg whites, (2) foods containing egg (e.g., baked goods, pancakes, mayonnaise), (3) peanut butter, and (4) peanuts.

### 2.4. Outcome—Age 3 Years

Among the 921 participants enrolled in the MARC-35 longitudinal cohort, the 582 (63%) suspected of having food allergy due to at least one of the following were selected for additional outcome assessment: (1) physician diagnosis or evaluation of food allergy on initial chart review up until age 0.9 years, (2) sensitization on either ImmunoCAP sIgE (≥0.1 kU/L) or ISAC (≥0.3 ISU) during infancy or the early childhood examination, (3) parent report of IgE-mediated symptoms (skin or breathing problems such as hives, swelling, itching, cough or wheezing) within two hours after eating at any long-term follow-up interview from age 12 months up until age 60 months, or (4) parent report of doctor-diagnosed IgE-mediated food allergy at any long-term follow-up interview from age 12 months up until age 60 months. We looked at interview data up until age 60 months rather than up until age 36 months to decrease the potential of missing food allergy cases. Among the 582 participants with suspected food allergy, further chart review was performed up until age 3 years and parents were surveyed via website or telephone regarding food allergy and dietary history.

Using all available data, IgE-mediated food allergy status by age 3 years for each participant was classified by allergists (E.Y., L.B.R., A.C.A.) as likely, possible, or unlikely using a standardized protocol (Figure S1). *Likely* food allergy represents the most definite cases of IgE-mediated food allergy. For example, a participant with parent report of doctor-diagnosed food allergy, testing highly suggestive of IgE-mediated food allergy, IgE-mediated symptoms with consumption of the food allergen, and subsequent avoidance of the food allergen would be categorized as *likely* food allergy. *Possible* food allergy represents less certain, but potential food allergic cases. For example, a participant with parent report of doctor-diagnosed food allergy, testing possibly suggestive of IgE-mediated food allergy, but no lifetime clinical exposure to the food, would be categorized as *possible* food allergy. *Unlikely* food allergy represents cases that are tolerant of food allergens. For example, participants with IgE-sensitization to a food allergen, but who do not have doctor-diagnosed food allergy and are able to consume the food would be categorized as *unlikely* to have food allergy. Remaining participants that did not meet criteria for additional food allergy evaluation were deemed unlikely to have IgE-mediated allergy to egg or peanut provided they had negative egg white and peanut sIgE (<0.35 kU/L) at infancy, and no report of either IgE-mediated symptoms after food ingestion or physician diagnosis of food allergy at the age 24 or 36 month interview. Of the 921 participants in the initial longitudinal cohort, one participant died during infancy, and 151 (16%) were excluded due to incomplete exposure data (e.g., age 12-month dietary history) or outcome data (e.g., age 24 or 36 month interview) (Figure S2).

### 2.5. Analysis

We obtained covariate data from the enrollment interview. Covariates of interest included sex, race/ethnicity, insurance provider, estimated median household income by ZIP code (Esri Business Analyst Desktop, Esri, Redlands, CA, USA), history of eczema at enrollment, parent with a history of food allergy, and number of other children living in the home.

All analyses were performed using Stata 15.1 (Stata Corp, College Station, TX, USA). Data are presented as proportions and medians with interquartile ranges. To evaluate associations between participant characteristics and early introduction of egg or peanut, we used chi-square, Fisher’s exact test, and Wilcoxon rank sum test, as appropriate. We used Fisher’s exact test to evaluate the association between early introduction of egg and development of likely egg allergy by age 3 years, and the association between early introduction of peanut and development of likely peanut allergy by age 3 years. All *p*-values were two-tailed, with *p* < 0.05 considered statistically significant.

## 3. Results

Of the 921 participants enrolled in the MARC-35 long-term follow-up cohort, 770 (84%) participants were included in the analytic cohort (Table 1, Figure S2). The median age at enrollment was 3.3 months. There were more male participants (*n* = 455, 59%) than female participants (*n* = 315, 41%). There were 356 (46%) non-Hispanic white, 163 (21%) non-Hispanic Black, and 223 (29%) Hispanic participants, and 28 (4%) participants identified as other race (including Asian and American Indian). Slightly more participants had public or no insurance (*n* = 433, 56%) compared to those with private insurance (*n* = 335, 44%), and approximately one-third lived in a ZIP code with median household income < USD 40,000 (*n* = 253, 33%). There were 109 (14%) participants with a reported history of eczema at enrollment, 151 (20%) with a reported parental history of food allergy, and the majority reported one or more other children living in the home (*n* = 601, 78%).

**Figure 1:**
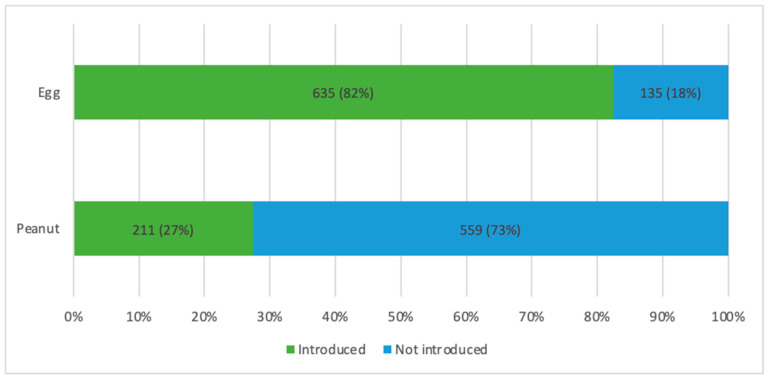
Introduction of egg and peanut at age 12 months.

Of the 770 participants in the analytic cohort, 635 participants (82%) reported regular consumption of egg product at 12 months of age, and 211 participants (27%) reported regular consumption of peanut product at 12 months of age (Table 1, Figure 1). There were no significant differences in age at enrollment, sex, or parental history of food allergy according to early introduction of egg or peanut (Table 1). However, participants without early introduction of egg were more likely to have a history of eczema at enrollment. Participants without early introduction of peanut were more often of non-Hispanic white ethnicity, with private insurance, median annual household incomes ≥ USD 40,000, and with fewer other children living in the home as compared to those with early introduction of peanut.

Bar graph depicting the number and percentage of participants with early introduction of egg as defined by parent report of egg product consumption at least once per week at age 12 months vs. those without (top bar) as well as the number and percentage of participants with early introduction of peanut as defined by parent report of peanut product consumption at least once per week at age 12 months vs. those without (bottom bar). Those with early introduction are depicted by green bars, and those without early introduction are depicted by blue bars.

In total, of the 770 participants in the analytic cohort, four (0.5%) participants had likely egg allergy by age 3 years, and eight (1%) participants had likely peanut allergy by age 3 years (1%) (Table 2). The cumulative incidence of likely egg allergy by age 3 years was 1/635 (0.2%) among subjects with early egg introduction and 3/135 (2.2%) among subjects without early egg introduction. Early introduction of egg prior to 12 months was associated with a lower incidence of IgE-mediated egg allergy by age 3 years. The cumulative incidence of likely peanut allergy by age 3 years was 0/211 (0%) among participants with early peanut introduction and 8/559 (1.4%) among subjects without early peanut introduction. For the participants with known dates of first allergic reaction to egg based on chart review, ages of first reaction ranged from 6 months (the one participant with early introduction of egg and egg allergy) to 21 months. For the participants with known dates of first allergic reaction to peanut based on chart review, ages of first reaction ranged from 18 months to 28 months.

Participants with likely vs. unlikely or possible food allergy to egg or peanut by age 3 years for the MARC-35 cohort, compared to those with and without early introduction of egg or peanut, respectively. Early introduction of egg or peanut are defined as consumption of egg product or peanut product, respectively, at least once per week at age 12 months. Results are shown as n (%). p-values less than 0.05 are shown in bold font.

## 4. Discussion

In this study of 770 infants with a history of severe bronchiolitis, 82% introduced egg by age 12 months, and only 27% introduced peanut by age 12 months. In total, four participants (0.5%) had likely egg allergy by age 3 years, and eight participants (1%) had likely peanut allergy by age 3 years. The cumulative incidence of likely egg allergy by age 3 years was 1/635 (0.2%) among subjects with early egg introduction and 3/135 (2.2%) among subjects without early egg introduction. The cumulative incidence of likely peanut allergy by age 3 years was 0/211 (0%) among participants with early peanut introduction and 8/559 (1.4%) among subjects without early peanut introduction. Early introduction of egg was associated with less egg allergy. Early introduction of peanut was suggestive of benefit as there were zero cases of allergy, but the association was not statistically significant.

While the majority of participants in our cohort had early introduction of egg, less than one-third of participants had early introduction of peanut. This is not necessarily unexpected given than the results of the major clinical trials in support of early introduction of egg and peanut emerged after our 2011–2014 study enrollment period. As previously highlighted, the results of the Learning Early about Peanut Allergy (LEAP) study were published in 2015 and showed that in a cohort of infants aged 4 to 11 months with egg allergy, severe eczema, or both, early introduction of peanut decreased the incidence of IgE-mediated peanut allergy [11]. In a follow-up two-group comparison study, the Persistence of Oral Tolerance to Peanut (LEAP-On) study showed that, among infants with early introduction of peanut and continued peanut consumption until 5 years of age, subsequent avoidance of peanut for 12 months was not associated with an increased risk of developing new peanut allergy [12]. The results of the Enquiring About Tolerance (EAT) study in 2016 showed, in an intention-to-treat analysis, that introduction of egg and peanut in breastfed infants by age 6 months was associated with a decreased prevalence of egg and peanut allergy by age 3 years [16].

Outside of the clinical trial setting, there are few real-world observational studies looking at the timing of egg or peanut introduction and the development of food allergy. The Australia-based cross-sectional study using the HealthNuts cohort found a higher risk of egg allergy associated with not introducing egg by age 6 months [19], similar to our findings of a higher risk of egg allergy associated with not introducing egg by age 12 months. Although our results for peanut were only suggestive, in a prospective Canadian birth cohort study, peanut introduction by age 12 months decreased the odds of peanut sensitization at age 12 months [31]. However, there are also clinical trials that failed to show a statistically significant association between introduction of egg by age 6 months and egg allergy [32,33,34] introduction of egg by age 8 months and egg allergy [35], or introduction of peanut by age 12 months and peanut allergy [36].

Although the gold standard for food allergy diagnosis is a double-blind placebo-controlled food challenge, there are several limitations to relying on food challenges, including time, expense, and burden on patients and families. Several notable food allergy studies have relied on alternative criteria such as parental report on questionnaires [37,38,39,40]. Another approach has been to use a combination of different methods including parental report, IgE sensitization, doctor diagnosis of food allergy, and epinephrine autoinjector prescription [17,41]. A unique strength of our study was the outcome ascertainment by an allergist’s review of multiple data sources including parental report on a food allergy survey, parent report on periodic interviews, review of medical records, and food specific IgE to determine whether IgE-mediated food allergy was likely.

The major limitation of our study was the small number of food allergy cases. This reduced our statistical power and precluded controlling for potential confounding factors in testing the association between early introduction and likely IgE-mediated food allergy. Importantly, eczema is a well-known confounder and effect modifier of egg and peanut allergy, and participants with early introduction of egg were less likely to have a history of eczema at enrollment when compared to those without early introduction of egg. The lower prevalence of eczema at enrollment may be related to the lower incidence of IgE-mediated egg allergy within this group [1,2,3,14,42,43,44,45,46]. Additionally, while similar to previous studies, prevalence of likely egg allergy and prevalence of likely peanut allergy within our cohort (0.5% and 1%, respectively) were lower than previously reported estimates in other US-based studies of food allergy in childhood of 0.8–0.9% for egg [37,38], and 1.4–2.2% for peanut [37,38,40,41]. Our lower prevalence of egg and peanut allergy may partially be explained by the heterogeneity among cohorts with different dietary patterns and food allergy risk factors as well as variation in the age groups studied. Additionally, the low number of participants with early introduction of peanut in this study, while not entirely unexpected in light of the time period in which our participants were enrolled, may have limited our ability to detect a significant association between early peanut introduction and decreased peanut allergy. An additional limitation of our study is that we used data from a severe bronchiolitis infant cohort, which may not necessarily be generalizable to the general population of US infants. However, bronchiolitis is not a rare childhood illness, given that the incidence of bronchiolitis hospitalizations among infants in the United States was 25.5 per 1000 children in 2011 [47]. The association between bronchiolitis and risk of food allergy is not known, but is of interest.

The present analysis adds to the literature by contributing real-world data in a racially/ethnically and geographically diverse cohort of US infants prior to updates to the food introduction guidelines. While clinical trials, especially of food introduction, are idealized, they are not often reproducible in a real-world setting. Additionally, food introduction has social and cultural ties, which may impact early food introduction and allergen exposure; this consideration supports the importance of studying a racially/ethnically diverse cohort. The real-world data from this study are important for helping researchers and clinicians better understand baseline dietary patterns and food allergy risk prior to revisions in infant feeding practice guidelines. Future comparative studies are needed to assess whether or not there has indeed been an increase in early introduction in clinical practice, and if so, if this increase has been effective in reducing food allergy prevalence.

## 5. Conclusions

In a cohort of infants hospitalized with bronchiolitis during 2011–2014, there were only four (0.5%) cases of likely egg allergy by age 3 years and eight cases of peanut allergy by age 3 years (1%). Although limited by small number of likely food allergy cases, our results—from a prospective cohort of geographically and racially/ethnically diverse infants—support the notion that early introduction of egg and peanut are associated with decreased risk of developing food allergy. These data lend support to recent major shifts in guidelines on infant food introduction practices. Future analyses are needed with larger numbers of food allergy cases for increased power to detect statistically significant and clinically meaningful differences. Additionally, we encourage validation of these findings in a healthy infant cohort.

## Figures and Tables

**Table 1:** Demographics and other characteristics of the analytic cohort at enrollment.

Characteristics	Analytic Cohort	Proportion with and without Early Introduction of Egg			Proportion with and without Early Introduction of Peanut		
	(*n* = 770)	Egg Introduced (*n* = 635, 82%)	Egg not Introduced (*n* = 135, 18%)	*p* Value	Peanut Introduced (*n* = 211, 27%)	Peanut not Introduced (*n* = 559, 73%)	*p* Value
Age at enrollment, months (median, IQR)	3.3 (1.6–6.0)	3.3 (1.6–5.9)	3.2 (1.6–6.3)	0.77	3.2 (1.5–5.7)	3.3 (1.7–6.1)	0.69
Sex (*n*, %)				0.47			0.38
Male	455 (59)	379 (60)	76 (56)		130 (62)	325 (58)	
Female	315 (41)	256 (40)	59 (44)		81 (38)	234 (42)	
Race/Ethnicity (*n*, %)				0.72			0.03
Non-Hispanic White	356 (46)	294 (46)	62 (46)		81 (38)	275 (49)	
Non-Hispanic Black	163 (21)	136 (21)	27 (20)		50 (24)	113 (20)	
Hispanic	223 (29)	184 (29)	39 (29)		74 (35)	149 (27)	
Other	28 (4)	21 (3)	7 (5)		6 (3)	22 (4)	
Insurance provider (*n*, %)				0.17			<0.001
Public or none	433 (56)	364 (58)	69 (51)		146 (70)	287 (51)	
Private	335 (44)	269 (43)	66 (49)		64 (30)	271 (49)	
Median household income by ZIP code (*n*, %)				0.50			<0.001
< USD 40,000	253 (33)	212 (33)	41 (30)		91 (43)	162 (29)	
≥ USD 40,000	517 (67)	423 (67)	94 (70)		120 (57)	397 (71)	
History of eczema	109 (14)	73 (12)	36 (27)	<0.001	25 (12)	84 (15)	0.26
Parental food allergy (*n*, %)	151 (20)	118 (19)	33 (24)	0.12	33 (16)	118 (21)	0.08
Other children living in the home (*n*, %)				0.43			0.005
0	169 (22)	135 (21)	34 (25)		42 (20)	127 (23)	
1	303 (39)	256 (40)	47 (35)		68 (32)	235 (42)	
≥2	298 (39)	244 (38)	54 (40)				0.38

**Abbreviations**: IQR, interquartile range. Demographics and characteristics of analytic cohort overall, and for those with and without early introduction of egg or peanut, as defined by consumption of peanut or egg product at least once per week at age 12 months. Data for participant characteristics were obtained at enrollment. Results are shown as n (%) unless otherwise noted. *p*-values less than 0.05 are shown in bold font.

**Table 2:** Early introduction of egg or peanut at age 12 months and likely food allergy to egg or peanut by age 3 years.

Food Allergy	Analytic Cohort *n* = 770	Early Introduction of Egg *n* = 635	No Early Introduction of Egg *n* = 135	*p* Value
				0.02
Egg allergy likely	4 (0.5)	1 (0.2)	3 (2.2)	
Egg allergy unlikely or possible	766 (99.5)	634 (99.8)	132 (97.8)	
	**Analytic Cohort** ***n* = 770**	**Early Introduction of Peanut** ***n* = 211**	**No Early Introduction of Peanut** ***n* = 559**	***p* Value**
				0.12
Peanut allergy likely	8 (1)	0 (0)	8 (1.4)	
Peanut allergy unlikely or possible	762 (99)	211 (100)	551 (98.6)	

## Data Availability

The data presented in this study are available on reasonable request from the corresponding author. The data are not publicly available due to privacy restriction.

## Supplementary Materials

The following are available online at https://www.mdpi.com/article/10.3390/nu13072318/s1, Figure S1: Allergist protocol for determination of likely, possible or unlikely food allergy, Figure S2: Flow diagram showing reasons for exclusion, Table S1: Food allergen components tested during infancy and at age 3 years, Table S2: Principal Investigators at the 17 participating sites in MARC-35.

## Abbreviations

EMNet	Emergency Medicine Network
EAT	Enquiring About Tolerance
ISAC	Immuno-solid-phase allergen chip
LEAP	Learning Early about Peanut Allergy
MARC	Multicenter Airway Research Collaboration
LEAP-On	Persistence of Oral Tolerance to Peanut
REDCap	Research Electronic Data Capture
sIgE	Specific-IgE
US	United States
