# Supplementary material for: Early Introduction of Food Allergens and Risk of Developing Food Allergy
Source: Nutrients. 2021 Jul 5;13(7):2318. doi: 10.3390/nu13072318 (PMC8308770; doi:10.3390/nu13072318)
Supplement: Supplementary file 1 [file nutrients-13-02318-s001.zip › nutrients-1260168-supplementary.pdf]

SUPPLEMENTARY MATERIAL

Early introduction of food allergens and risk of developing food allergy

Elizabeth Yakaboski, MD[1]; Lacey B. Robinson, MD, MPH[1]; Anna Chen Arroyo, MD, MPH[2]; Janice A. Espinola, MPH[3]; Ruth J. Geller, MHS[3]; Ashley F. Sullivan, MPH[3]; Susan A. Rudders, MD[4]; Carlos A. Camargo, Jr., MD, DrPH[1,3]

Contact info:
Carlos A. Camargo, MD, DrPH, Department of Emergency Medicine, Massachusetts General Hospital, 125 Nashua St, Suite 920, Boston, MA 02114.
Phone: 617-726-5276 Fax: 617-724-4050 Email: ccamargo@partners.org

Table of Contents:

- **Figure S1:** Allergist protocol for determination of likely, possible or unlikely food allergy
- **Figure S2:** Flow diagram showing reasons for exclusion
- **Table S1: Food allergen components tested during infancy and at age 3 years**
- **Table S2:** Principal Investigators at the 17 participating sites in MARC-35

Figure S1: Allergist protocol for determination of likely, possible or unlikely food allergy

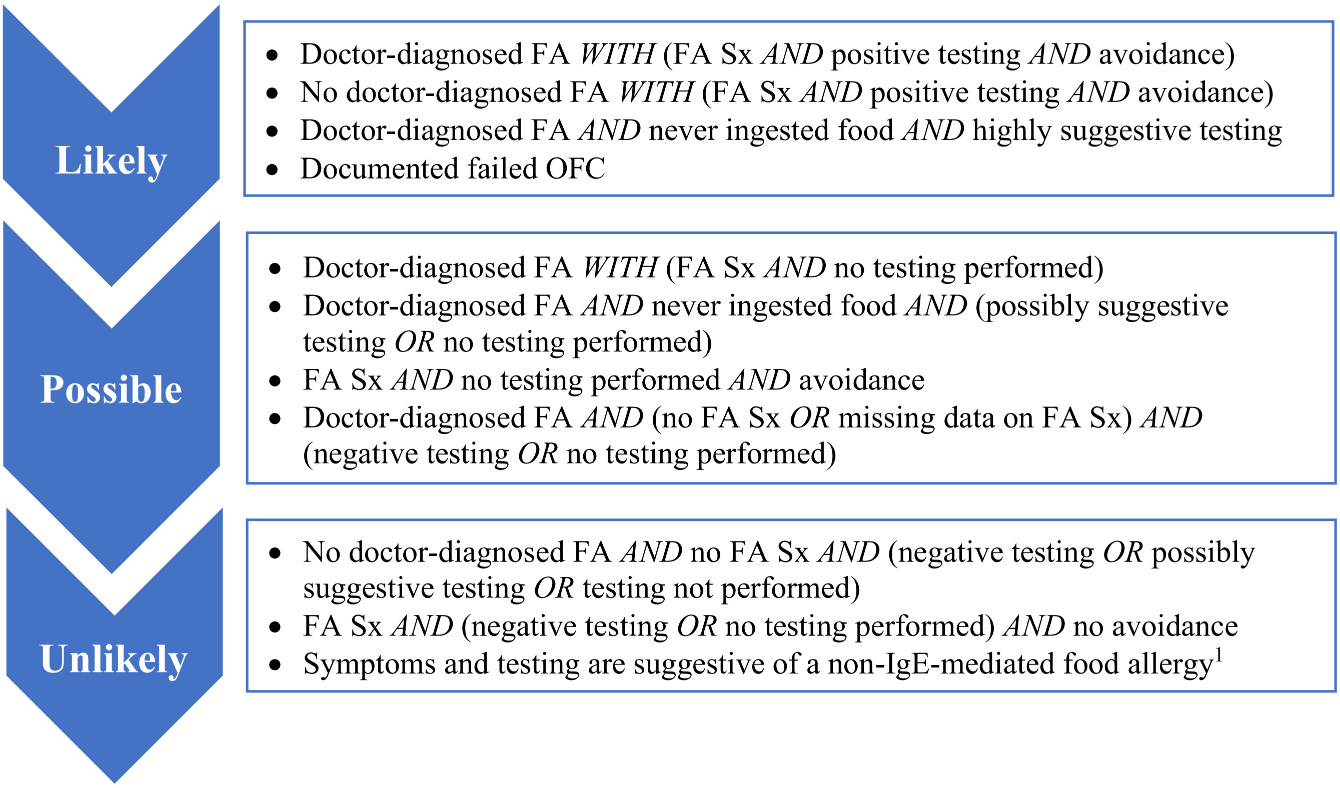

Abbreviations: FA, food allergy; FA Sx, food allergy symptoms; OFC, oral food challenge

[1] E.g., lactose intolerance, food protein-induced allergic proctocolitis (FPIAP), food protein-induced enterocolitis syndrome (FPIES), and celiac disease.

Allergist protocol for determination of likely, possible or unlikely food allergy based on clinical scenario. Doctor-diagnosed food allergy and food allergy symptoms may be per parent report or per medical record documentation. For "likely" food allergy based on criteria including doctor-diagnosed food allergy, reviewing allergist must agree with the diagnosis. Food allergy symptoms refers to IgE-mediated symptoms within two hours of food allergen exposure. IgE-mediated symptoms includes hives, angioedema, wheezing, vomiting, and diarrhea. Positive testing refers to positive skin prick test and/or IgE $\geq$ 0.35 kU/L; possibly suggestive testing refers to testing that is positive, but associated with a positive predictive value of $<$ 95%; testing that is highly suggesting refers to positive testing that is associated with a positive predictive value of $\geq$ 95%. Avoidance refers to dietary avoidance of a potential food allergen due to concern for food allergy. No avoidance refers to subsequent ingestion of food per parent report or medical records.

Figure S2: Flow diagram showing reasons for exclusion

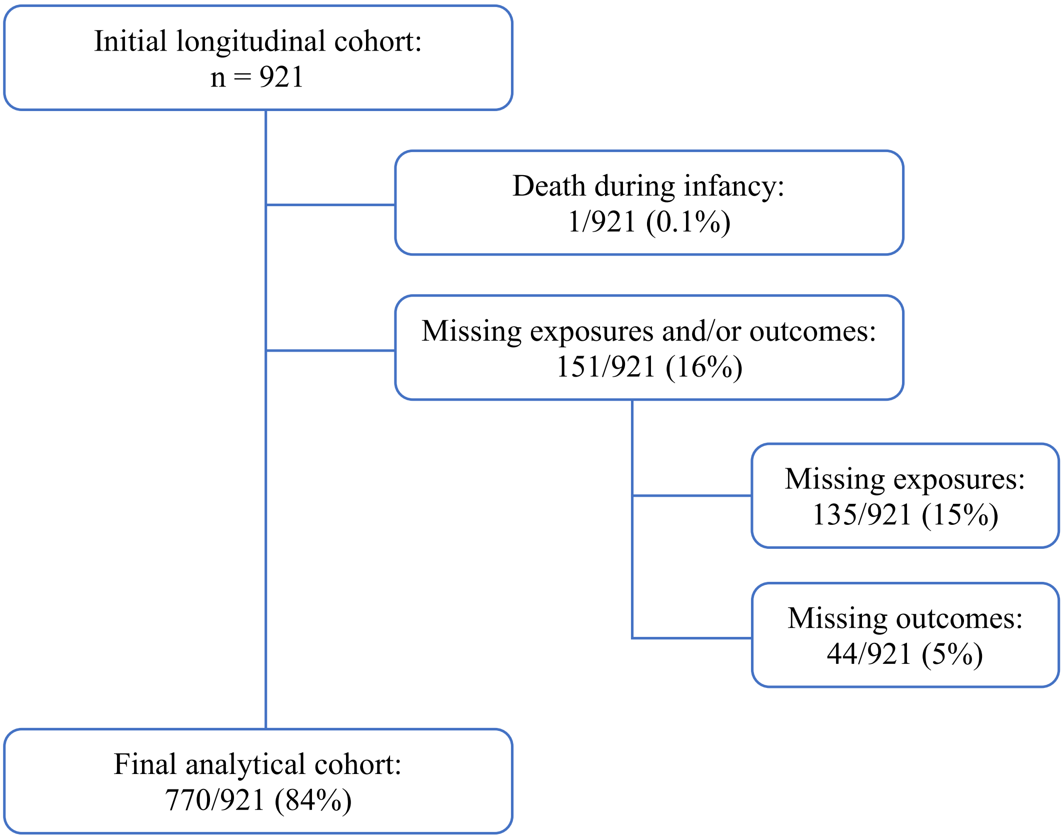

Flow diagram illustrating number of participants in the initial longitudinal cohort, number and percentage of participants excluded, and number and percentage of participants included in the final analytical cohort. One participant was excluded due to death during infancy and remaining participants were excluded due to missing exposures and/or outcomes.

Table S1: Food allergen components tested during infancy and at age 3 years

Immuno-solid-phase allergen chip (ISAC)			
**Allergen**	**Component**	**Allergen**	**Component**
**Egg white**	Gal d 1	**Cod**	Gad c 1
	Gal d 2	**Brazil nut**	Ber e 1
	Gal d 3	**Hazelnut**	Cor a 1.0401
**Cow's milk**	Bos d 4		Cor a 9
	Bos d 5		Cor a 8
	Bos d 6	**Sesame seed**	Ses i 1
	Bos d 8	**Soybean**	Gly m 4
	Bos d lactoferrin		Gly m 5
**Cashew**	Ana o 2		Gly m 6
**Walnut**	Jug r 1	**Buckwheat**	Fag e 2
	Jug r 2	**Wheat**	Tri a 14
	Jug r 3		Tri a 19.0101
**Peanut**	Ara h 1		Tri a aA_TI
	Ara h 2	**Kiwi**	Act d 1
	Ara h 3		Act d 2
	Ara h 6		Act d 5
	Ara h 8		Act d 8
	Ara h 9	**Peach**	Pru p 1
**Egg yolk/chicken**	Gal d 5		Pru p 3
**Shrimp**	Pen m 1	**Apple**	Mal d 1
	Pen m 2	**Celery**	Api g 1
	Pen m 4	**Bromelain**	MUXF3

Table detailing the food allergens and respective components on the Immuno-solid-phase allergen chip assay obtained during infancy and at age 3 years. In addition to the component testing above, five whole food allergens were tested via ImmunoCAP specific-IgE: egg white, cow’s milk, cashew nut, walnut, and peanut.

Table S2: Principal Investigators at the 17 participating sites in MARC-35

Amy D. Thompson, MD	Alfred I. duPont Hospital for Children, Wilmington, DE
Federico R. Laham, MD, MS	Arnold Palmer Hospital for Children, Orlando, FL
Jonathan M. Mansbach, MD, MPH	Boston Children's Hospital, Boston, MA
Vincent J. Wang, MD, MHA and Susan Wu, MD	Children's Hospital of Los Angeles, Los Angeles, CA
Michelle B. Dunn, MD and Jonathan M. Spergel, MD, PhD	Children's Hospital of Philadelphia, Philadelphia, PA
Juan C. Celedon, MD, DrPH	Children's Hospital of Pittsburgh, Pittsburgh, PA
Michael R. Gomez, MD, MS-HCA, Nancy R. Inhofe, MD	Children's Hospital at St. Francis, Tulsa, OK
Brian M. Pate, MD and Henry T. Puls, MD	Children's Mercy Hospital & Clinics, Kansas City, MO
Stephen J. Teach, MD, MPH	Children's National Medical Center, Washington, D.C.
Stephen C. Porter, MD, MSc, MPH and Richard T. Strait, MD	Cincinnati Children's Hospital and Medical Center, Cincinnati, OH
Ilana Y. Wanik, MD	Connecticut Children's Medical Center, Hartford, CT
Sujit S. Iyer, MD	Dell Children's Medical Center of Central Texas, Austin, TX
Ari R. Cohen, MD, Margaret Samuels-Kalow, MD, MPhil, MSHP and Wayne G. Shreffler, MD, PhD	Massachusetts General Hospital, Boston, MA
Michelle D. Stevenson, MD, MS	Norton Children's Hospital and the University of Louisville, Louisville, KY
Cindy S. Bauer, MD and Anne K. Beasley, MD	Phoenix Children's Hospital, Phoenix, AZ
Markus Boos, MD, PhD and Thida Ong, MD	Seattle Children's Hospital, Seattle, WA
Charles G. Macias, MD, MPH and Sarah Meskill, MD	Texas Children's Hospital, Houston, TX

Principal Investigators at the 17 participating sites in MARC-35, in order of their affiliated institution.
